# Incorrect strain information for mouse cell lines: sequential influence of misidentification on sublines

**DOI:** 10.1007/s11626-016-0104-3

**Published:** 2016-11-14

**Authors:** Kozue Uchio-Yamada, Fumio Kasai, Midori Ozawa, Arihiro Kohara

**Affiliations:** 1 Laboratory of Animal Models for Human Diseases, National Institutes of Biomedical Innovation, Health and Nutrition, 7-6-8 Saito-Asagi, Ibaraki Osaka, 567-0085 Japan; 2 Japanese Collection of Research Bioresources (JCRB) Cell Bank, Laboratory of Cell Cultures, National Institutes of Biomedical Innovation, Health and Nutrition, 7-6-8 Saito-Asagi, Ibaraki Osaka, 567-0085 Japan

**Keywords:** Cell culture, Cell line profile, Strain identification, Simple sequence length polymorphism (SSLP)

## Abstract

Misidentification or cross-contamination of cell lines can cause serious issues. Human cell lines have been authenticated by short tandem repeat profiling; however, mouse cell lines have not been adequately assessed. In this study, mouse cell lines registered with the JCRB cell bank were examined by simple sequence length polymorphism (SSLP) analysis to identify their strains. Based on comparisons with 7 major inbred strains, our results revealed their strains in 80 of 90 cell lines. However, 12 of the 80 cell lines (15%) were found to differ from registered information. Of them, 4 cell lines originated from the same mouse, which had been generated through mating between two different inbred strains. The genotype of the mouse sample had not been examined after the backcross, leading to strain misidentification in those cell lines. Although 8 other cell lines had been established as sublines of a BALB/c cell line, their SSLP profiles are similar to a Swiss cell line. This affects differences in genotypes between inbred and outbred strains. Because the use of inbred samples and interbreeding between strains are not involved in human materials, our results suggest that the cause and influence of misidentification in mouse cell lines are different from those in human.

**Electronic supplementary material:**

The online version of this article (doi:10.1007/s11626-016-0104-3) contains supplementary material, which is available to authorized users.

## Introduction

Misidentification or cross-contamination of cell lines is a serious problem, leading to erroneous research (American Type Culture Collection Standards Development Organization Workgroup ASN-0002 2010; Capes-Davis et al. 2010). This problem is caused by poor technique in handling cell lines and a lack of routine quality control. To eliminate misidentified or cross-contaminated cell lines, various techniques have been employed. Karyotyping and isoenzyme techniques can reveal interspecies genetic differences (Ono et al. 2007), and profiling of short tandem repeat (STR) polymorphisms is a robust method for detection of intraspecies misidentification.

Human cell lines distributed through ATCC, DSMZ, RIKEN, and JCRB repositories are authenticated by STR analysis, and these cell banks have established comprehensive STR databases of human cell lines (Masters et al. 2001; Barallon et al. 2010; Dirks et al. 2010; Capes-Davis et al. 2013). Although mouse cell lines are also used for a wide range of research fields, their authentication has not yet been fully explored, partly because the majority of lines originated from a limited number of inbred strains which cannot be discriminated by conventional STR analysis.

Mouse genetic background influences the susceptibility to various diseases, including cancer and infectious diseases (Wang et al. 2004; Anh et al. 2006). In the generation of tumor-bearing mice utilized by cell transplantation, it is important to use the same strain between hosts and injected cells. These points imply that misidentification of mouse strains may lead to different experimental results. The RIKEN BioResource Center has developed a practical method to determine the mouse strain based on simple sequence length polymorphism (SSLP) analysis using 6 microsatellite markers and applies this to identify the derived strain of mouse cell lines (Yoshino et al. 2010). Using this method, we investigated the incidence of strain misidentification of mouse cell lines in the JCRB cell bank.

## Materials and Methods

### Mouse cell lines and inbred mouse strains

A list of cell lines examined in this study is shown in Table 1. These cell lines are registered with the JCRB cell bank as *Mus musculus* origin and distributed upon request. Information about each cell line is available through the JCRB website (http://cellbank.nibiohn.go.jp/english). C57BL/6, BALB/c, DBA/2, 129, and C3H mice obtained from CLEA Japan (Tokyo, Japan), A and CBA from Japan SLC (Hamamatsu, Japan) were used as reference panels. All animal experiments were performed in accordance with protocols approved by the Institutional Animal Care and Use Committees of National Institutes of Biomedical Innovation, Health and Nutrition.

**Table 1.** Strains of mouse cell lines identified by SSLP analysis

Registered strain	Number of cell lines	Results				
		Strain	Matched registered strain	Different from registered strain	Newly identified in this study	Unidentified
129	2	129	2			
C57BL/6	14	C57BL/6	9			
		C57BL/6 and CBA		4		
C3H	18	C3H	18			
DBA/2	4	DBA/2	4			
BALB/c	31	BALB/c	23			
		Swiss		8		
A	1	A	1			
BALB/c × DBA/2	1	BALB/c × DBA/2	1			
BALB/c × C57BL/6	2	BALB/c × C57BL/6	2			
Swiss	5	Swiss	5			
B6C3 F1	1	–				1
C57L	1	–				1
LAF1	2	–				2
ICR	1	–				1
ddN	1	–				1
CFW	1	–				1
SL	1	–				1
Unknown	5	BALB/c			1	
		C3H			1	
		DBA/2			1	
		–				2
Total	90		65	12	3	10

### DNA preparation and SSLP analysis

Genome DNA was isolated from approximately 5 × 10^6^ cells using AllPrep DNA/RNA Mini Kit (QIAGEN, Hilden, Germany) or from a short piece of mouse tail using DNeasy Blood and Tissue Kit (QIAGEN). SSLP analysis was carried out at 7 loci using primer sequences available from the Mouse Microsatellite Data Base of Japan (http://shigen.nig.ac.jp/mouse/mmdbj) listed in Table S1. The 5′ end of the sense primers was labeled with Beckman Dye (Sigma, St. Louis MO). PCR was performed using the Go Taq Green Master Mix (Promega, Madison WI) for 35 cycles of 94°C for 30 s, 55°C for 30 s, and 72°C for 30 s, followed by a final extension at 72°C for 7 min. After amplification, samples were electrophoresed and analyzed by CEQ8800 Genetic Analysis System (Beckman Coulter, Brea CA).

## Results and Discussion

**Figure 1.**
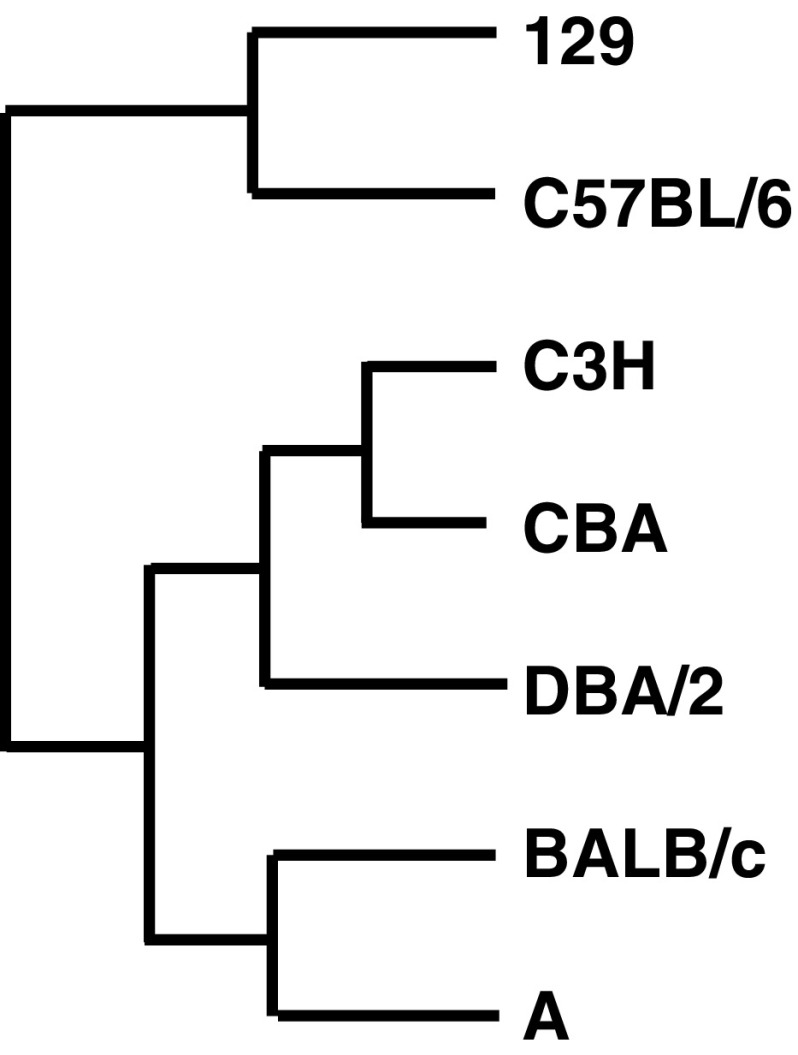
Relationship of 7 inbred strains used for reference SSLP profiles in this study. Phylogenetic tree is based on a previous study by Witmer et al. (2003). Strains 129 and C57BL/6 belong to a different branch from other strains, which is consistent with our reference data.

Reference SSLP sizes for 6 loci in the 6 inbred strains (Tables 2 and 3) show differences in markers between strains (Table 4, Fig. S1), indicating that combination of the 6 SSLP loci can distinguish among the 6 inbred strains. Comparison of the profiles between strains reveals that the shortest and largest sizes can be distinct markers to identify the strain (Fig. S2). The largest and shortest sizes detected at D1Mit159 and D13Mit253 loci, respectively, are characteristics of the C57BL/6 strain. Although 2 of 6 markers at D4Mit170 and D5Mit357 are identical between the C57BL/6 and 129 strains, the latter has the largest size at the D2Mit395 locus. BALB/c and DBA/2 have a distinctive size at D1Mit159 and share a common profile at 4 of 6 loci; however, the shortest size at D5Mit357 is unique to BALB/c. C3H can be characterized by the shortest size at the D17Mit51 locus. The A strain shares 2 loci with C3H and another 2 loci with DBA/2 and BALB/c. These similarities indicate that the 6 strains can be largely distinguished by two lineages consisting of (C57BL/6 and 129) and (BALB/c, DBA/2, C3H, and A). This is consistent with the phylogenetic tree based on 314 SSLP markers (Fig. 1, Witmer et al. 2003). Our evaluation of the 6 SSLP markers shows that they are qualified for identification of the 6 inbred strains.

**Table 2.** SSLP reference data of common mouse inbred strains

Marker	D1Mit159	D2Mit395	D4Mit170	D5Mit357	D13Mit253	D17Mit51
129	180	*154*	103	124	105	163
C57BL/6	*190*	126	103	124	*77*	157
C3H	174	120	113	144	101	*140*
A	174	133	103	130	101	154
DBA/2	*134*	133	119	144	98	154
BALB/c	*134*	133	119	*114*	109	154

Italics indicate the shortest and largest sizes, which are distinct from other strains and can be markers to identify the strain

**Table 3.** SSLP reference data to distinguish CBA from C3H

Marker	D4Mit196
C3H	200
CBA	187

**Table 4.** Number of polymorphic markers based on 6 loci between 6 inbred mouse strains

	BALB/c	DBA/2	A	C3H	C57BL/6
129	6	6	5	6	4
C57BL/6	6	6	5	6	
C3H	6	5	4		
A	4	4			
DBA/2	2

Our SSLP profiles of mouse cell lines were compared with the reference data to identify the strains of their origins (Table S2), summarized in Table 1. It was confirmed that 60 cell lines were correctly registered with their strain information, including 3 cell lines originated from hybrids between two inbred backgrounds. Strains of 3 cell lines previously unknown are clarified in this study. In total, 60 cell lines have been established from inbred mouse strains, which exhibit a single marker size in each locus except for 2 cell lines, IKK-i-DEF and TBK-i-DEF, originated from gene-deficient mice. Analysis of 5 cell lines registered as Swiss origin shows highly similar profiles between them. As Swiss is not an inbred strain, more than two different alleles are detected at 4 of 6 loci in 3 of the 5 cell lines, 3T3 L1 (JCRB9014), 3T3-Swiss albino (JCRB9019), and 3T3-L1 (IFO50416), which are closer to the origin than other two sublines. Instead of a reference sample obtained from a Swiss individual, 3T3 L1 cell line (JCRB9014) corresponding to ATCC® CL-173™ is used for the Swiss reference. Because of a limited number of references, 10 cell lines without strain information could not be validated within the 6 inbred strains. Two different alleles are observed in 8 of them, implying that those cell lines would be originated from outbred strains. Three alleles detected at D5Mit357 in Ehrlich ascites (JCRB9090) could be caused by genome instability of tumor cells because this cell line is reported to be aneuploid (Nielsén 1967).

Four cell lines (JCRB1198.1, JCRB1199, JCRB1225, and JCRB1207) had been deposited as of C57BL/6 origin. However, SSLP profiles of these cell lines show mixed profiles between C57BL/6 and C3H, indicating that they were derived from an intercrossed individual (Table 5). It is reported that they were established from a beta-galactosidase knockout mouse produced from an ES cell line TT2 from an F1 embryo between a C57BL/6 female and a CBA male (Matsuda et al. 1997; Tominaga et al. 2001). Our inquiry to the developers revealed that the knockout mouse was not backcrossed to C57BL/6 when they established the cell lines. Because C3H and CBA are closely related strains and the 6 SSLP markers are identical between them, another marker, D4Mit196, is added to identify their strains (Table 3). The additional reference locus shows the differences between the two strains, leading to correction of their strain information.

**Table 5.** Strain misidentification in mouse cell lines revealed by SSLP analysis

JCRB No.	Cell name	D1Mit159	D2Mit395	D4Mit170	D5Mit357	D13Mit253	D17Mit51	D4Mit196	Registered strain	Result
JCRB1198.01	GP8	190	120	103	143	101	142	187	C57BL/6	C57BL/6 and CBA
JCRB1199	R201C	190	120	103	144	101	142	187	C57BL/6	C57BL/6 and CBA
JCRB1225	I51T	190	120	103	143	101	142	187	C57BL/6	C57BL/6 and CBA
JCRB1207	SV	190	120	103	143	101	142	187	C57BL/6	C57BL/6 and CBA
IFO50070	BALB/3T3 A31-1-1	179	119/157	103	113/143	97/110	141	–	BALB/c	Swiss
IFO50298	Balb/c 3T3 A31-I-1	179	119/157	103	113/144	110	141	–	BALB/c	Swiss
IFO50299	Balb/c 3T3 A31-1-13	179	119/157	103	113/144	110	141	–	BALB/c	Swiss
JCRB0601	BALB/3T3 A31–1-1	179	119/157	103	113/143	97/110	141	–	BALB/c	Swiss
JCRB0149	Bhas42	179	119	103	113/143	97/110	141	–	BALB/c	Swiss
JCRB1355	1-1ras1000	179	119/157	103	113/143	97/110	141	–	BALB/c	Swiss
JCRB1356	A31-1-1	179	119/157	103	113/143	97/110	141	–	BALB/c	Swiss
JCRB1357	1-1src	179	119/157	103	113/143	97/110	141	–	BALB/c	Swiss

**Figure 2.**
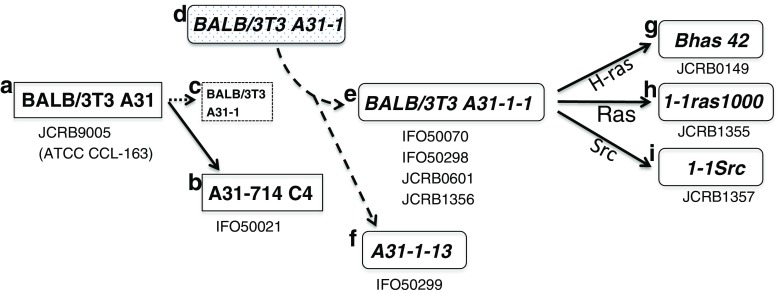
Strain misidentification occurred in BALB/3T3 A31 related mouse cell lines. (*a*) BALB/3T3 A31 (JCRB9005) was established from a BALB/c mouse. (*b*) A31-714 C4 (IFO50021) is one of the sublines generated from BALB/3T3 A31. These two cell lines are confirmed to be BALB/c strain by SSLP analysis in this study. (*c*) BALB/3T3 A31-1 cell line was reported as a subline of BALB/3T3 A31 but this is not registered at any cell banks. (*d*) Misidentification occurred in the BALB/3T3 A31-1 cell line, which had been replaced with a Swiss cell line. (*e*, *f*) BALB/3T3 A31-1-1 and A31-1-13 were established from the misidentified cell line. (*g*–*i*) Bhas 42, 1-1ras1000, and 1-1Src were generated from the *Swiss* BALB/3T3 A31-1-1 cell line but have been believed to be inbred BALB/c strain. This led to strain misidentification involving 8 mouse cell lines registered with the JCRB cell bank.

Eight cell lines had been believed as subclones of the BALB/3T3 clone A31 cell line derived from the BALB/c strain (Kakunaga and Crow 1980; Sasaki et al. 1988, 1990; Tatsuka et al. 1996, 1997). However, SSLP analysis of these 8 samples showed 2 different lengths for 2 or 3 loci, indicating that they did not originate from an inbred strain (Table 5). Although BALB/3T3 A31 and A31-714 C4 cell lines have been confirmed as BALB/c strain, the other 8 cell lines have profiles similar to the 3T3 L1 cell line, indicating that they were derived from a Swiss albino strain (Fig. 2). It is reported that BALB/3T3 A31-1-1 and A31-1-13 were established from BALB/3T3 A31-1, a subclone of BALB/3T3 A31 (Kakunaga and Crow 1980). However, in our survey, there is no BALB/3T3 A31-1 subline originating from BALB/c strain. This suggests that BALB/3T3 A31-1-1 had been established from a misidentified cell line originating from a Swiss strain and does not exist as a subline of BALB/3T3 A31. Three cell lines, Bhas 42, 1-1ras1000, and 1-1Src, have been established from BALB/3T3 A31-1-1 and can be explained with wrong strain information. It is possible to perform comparative analysis between these 8 cell lines under the same genetic background. Because of differences in susceptibility to chemicals and viral infections between BALB/c and Swiss mice (Nazarov et al. 1994; Wang et al. 2004), it is noted that these data cannot be used for a straightforward approach to compare with the BALB/3T3 A31 cell line.

It is reported that the STR-based multiplex assay could distinguish between three BALB/c-derived cell lines by one repeat unit (Almeida et al. 2014). However, the differences are not significant to characterize each cell line because genetic components between cell lines carrying the same name can be changed during cell culture (Kasai et al. 2016). This can be found in the human STR database, showing that all STR loci are not always identical between the same cell lines registered at different cell banks. Although SSLP profiling based on MIT markers lacks the resolution to discriminate between cell lines originating from the same mouse strain, it has been established for in vivo experiments to identify strain when congenic mice are generated through backcross (Markel et al. 1997). This approach can be used as a conventional technique to detect interstrain misidentification and is an efficient method for reducing misidentification in mouse cell lines.

Each mouse strain has different genomic characteristics and an appropriate mouse strain is carefully selected for in vivo experiments, to fulfill each research purpose, suggesting that strain misidentification in mouse cell lines could lead to misleading results. Because misidentification can easily occur during routine experiments, it is essential for all cell lines to be characterized by genetic markers such as STR, SSLP, or single nucleotide polymorphism (SNP) before they are used. SNP analysis has been developed for authentication of mouse cell lines (Didion et al. 2014); however, this genotyping array has not been used as a standard method. Because inbred materials are not used in human cell lines, our results show differences in misidentification between human and mouse cell lines. Strain identification in mouse cell lines plays an important role in the use of mouse cell lines as in vitro models.

## Electronic supplementary material

Figure S1 (PDF 608 kb)

Figure S2 (PDF 120 kb)

Table S1 (XLSX 10 kb)

Table S2 (XLSX 18 kb)
