# Supplementary material for: Incorrect strain information for mouse cell lines: sequential influence of misidentification on sublines
Source: In Vitro Cell Dev Biol Anim. 2016 Nov 14;53(3):225–30. doi: 10.1007/s11626-016-0104-3 (PMC5348555; doi:10.1007/s11626-016-0104-3)

Figure S1

Examples of electropherograms from SSLP analysis based on MIT markers. Inbred strains, C57BL/6 and BALB/c, show one main peak in each locus. The peak distribution pattern of GP8 (JCRB1198.01) also exhibits apparently inbred strain but is different from C57BL/6, provided information from the originator when deposited. BALB/3T3 A31-1-1 (JCRB0601) and 3T3-L1 (JCRB9014) show highly similar profiles with 2 main peaks for 3 or 4 locus, indicating that they originate from outbred strains.

BALB/c

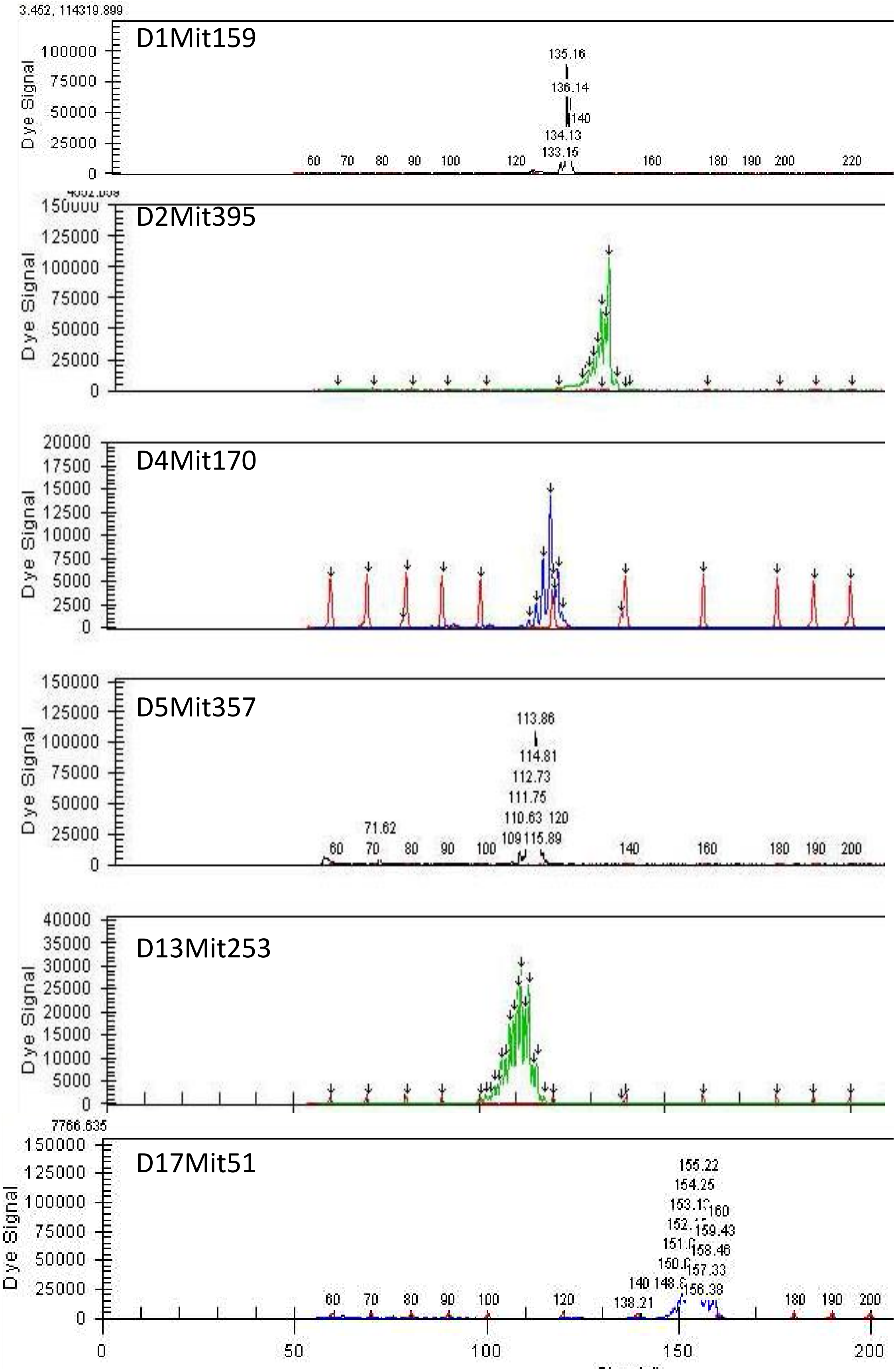

3.452, 114319.899
D1Mit159
135.16
136.14
140
134.13
133.15
D2Mit395
D4Mit170
D5Mit357
113.86
114.81
112.73
111.75
110.63
120
109
115.89
71.62
D13Mit253
7766.635
D17Mit51
155.22
154.25
160
159.43
158.46
157.33
156.38
138.21
Dye Signal
0
50
100
150
200

C57BL/6

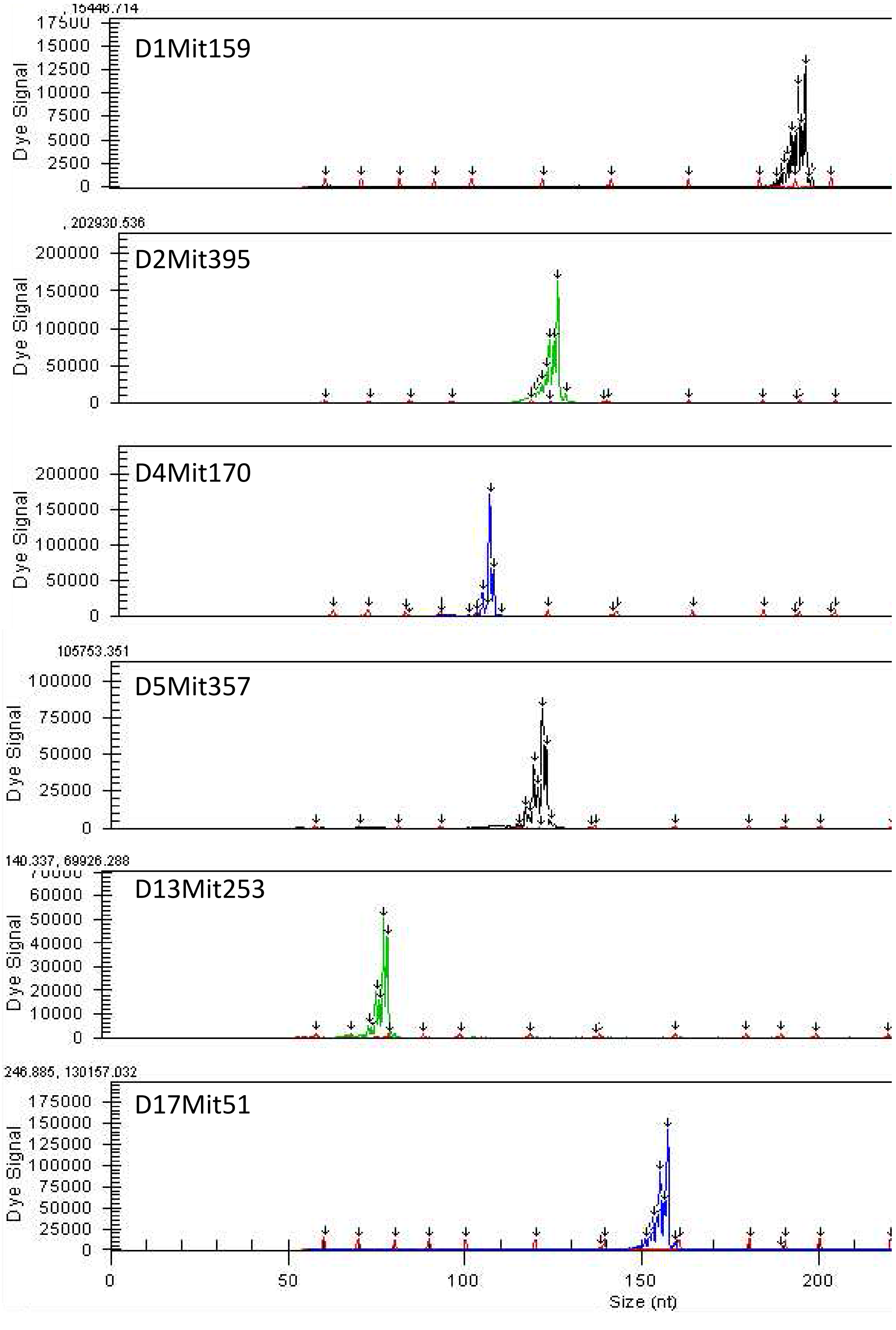

D1Mit159
D2Mit395
D4Mit170
D5Mit357
D13Mit253
D17Mit51
Dye Signal
Size (nt)

JCRB9014 3T3-L1

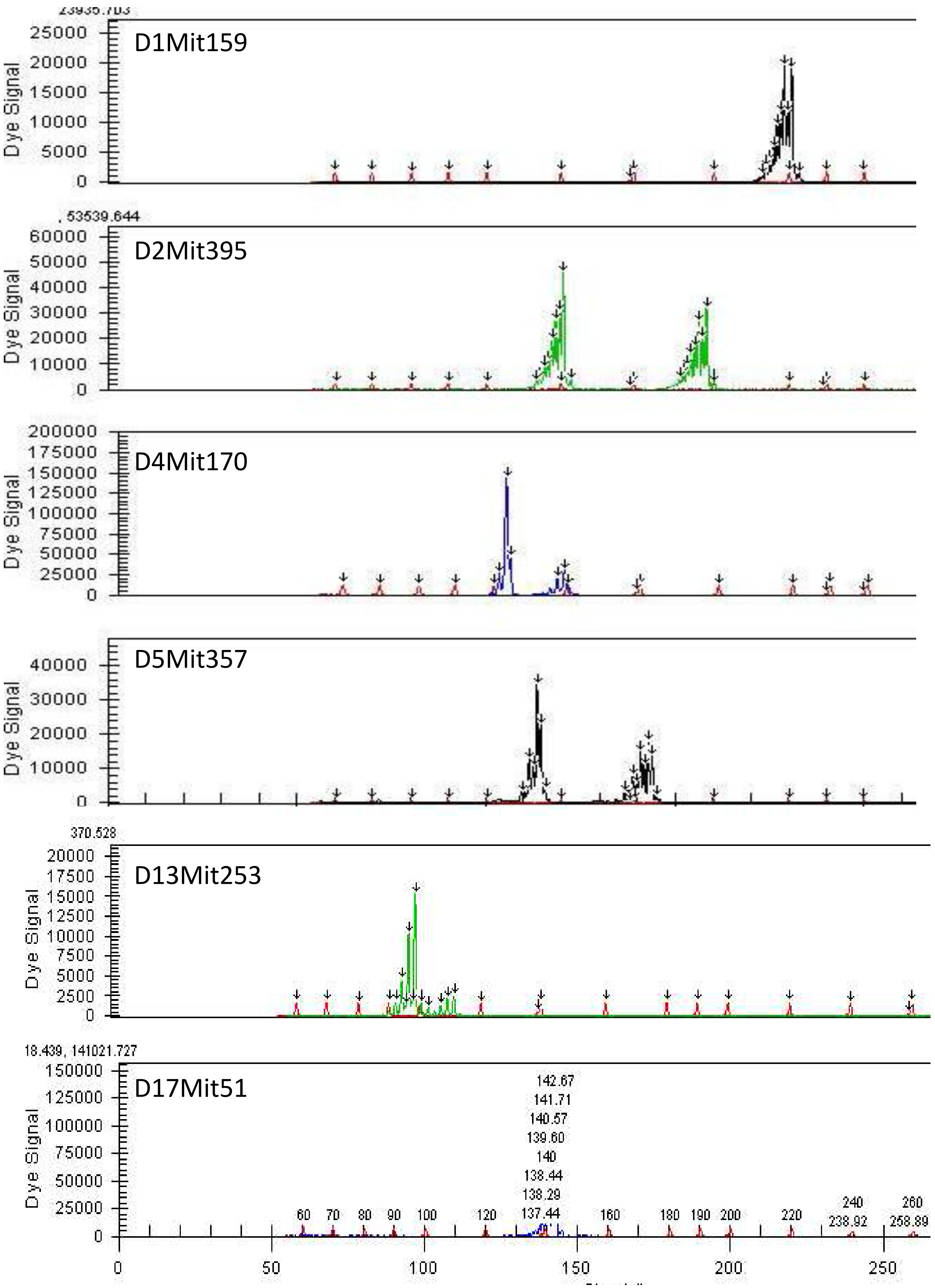

JCRB0601 BALB/3T3 A31-1-1

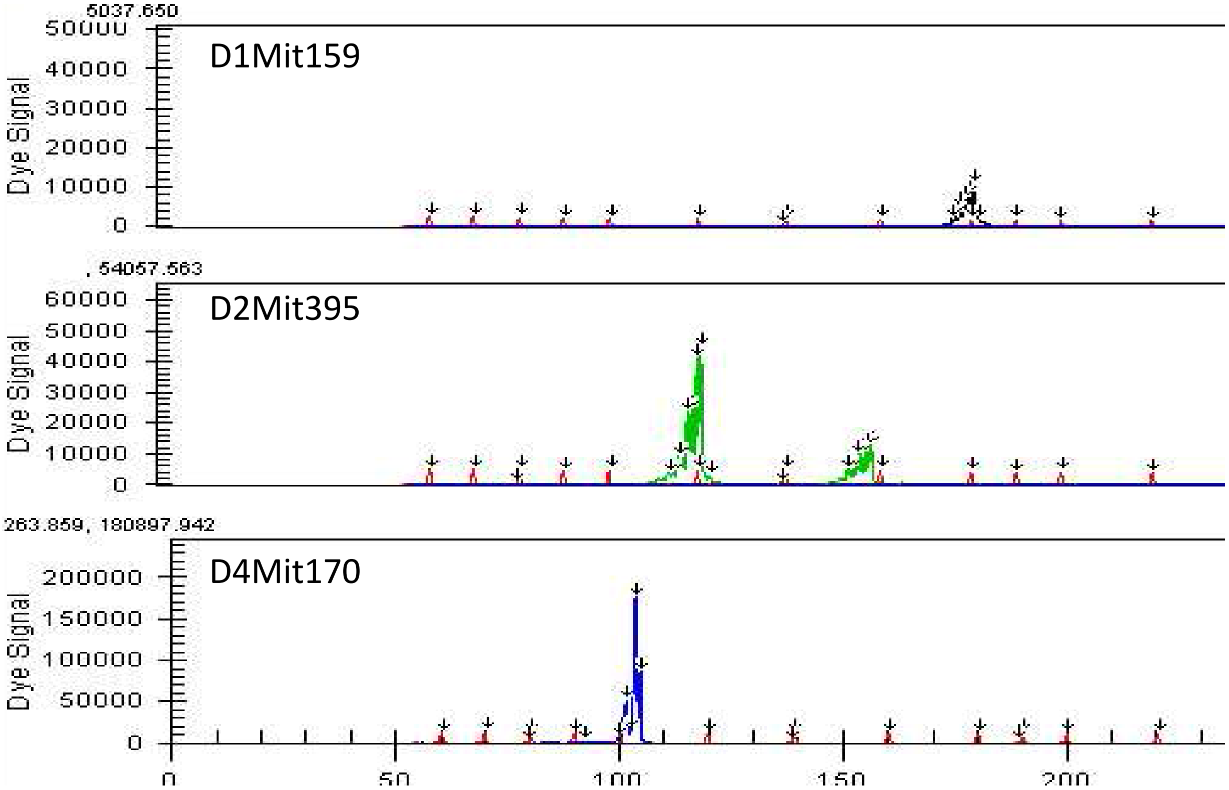

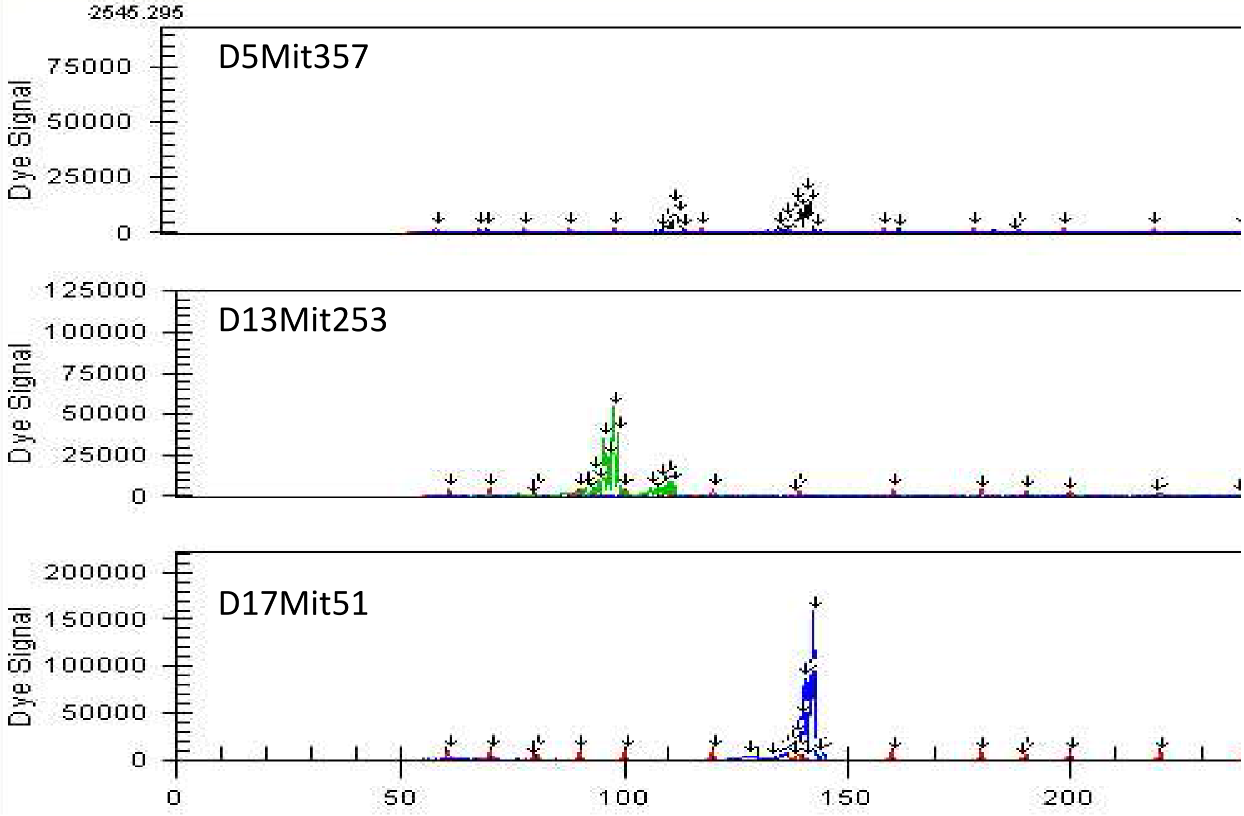

JCRB1198.01 GP8

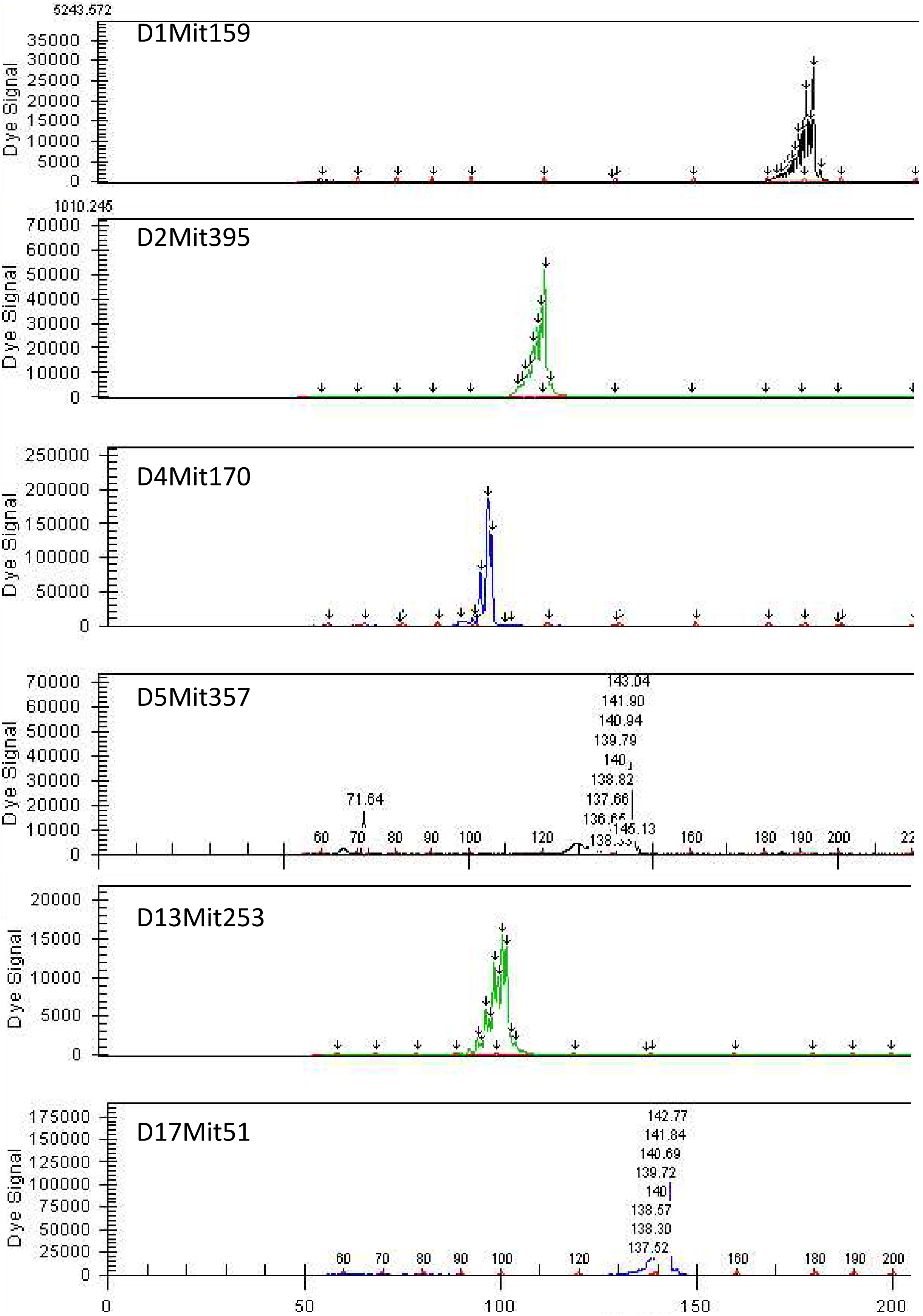

Supplement: Supplementary file 1 — (PDF 608 kb) [file 11626_2016_104_MOESM1_ESM.pdf]
