# Supplementary material for: Incorrect strain information for mouse cell lines: sequential influence of misidentification on sublines
Source: In Vitro Cell Dev Biol Anim. 2016 Nov 14;53(3):225–30. doi: 10.1007/s11626-016-0104-3 (PMC5348555; doi:10.1007/s11626-016-0104-3)
Supplement: Supplementary file 2 — (PDF 120 kb) [file 11626_2016_104_MOESM2_ESM.pdf]

Figure S2

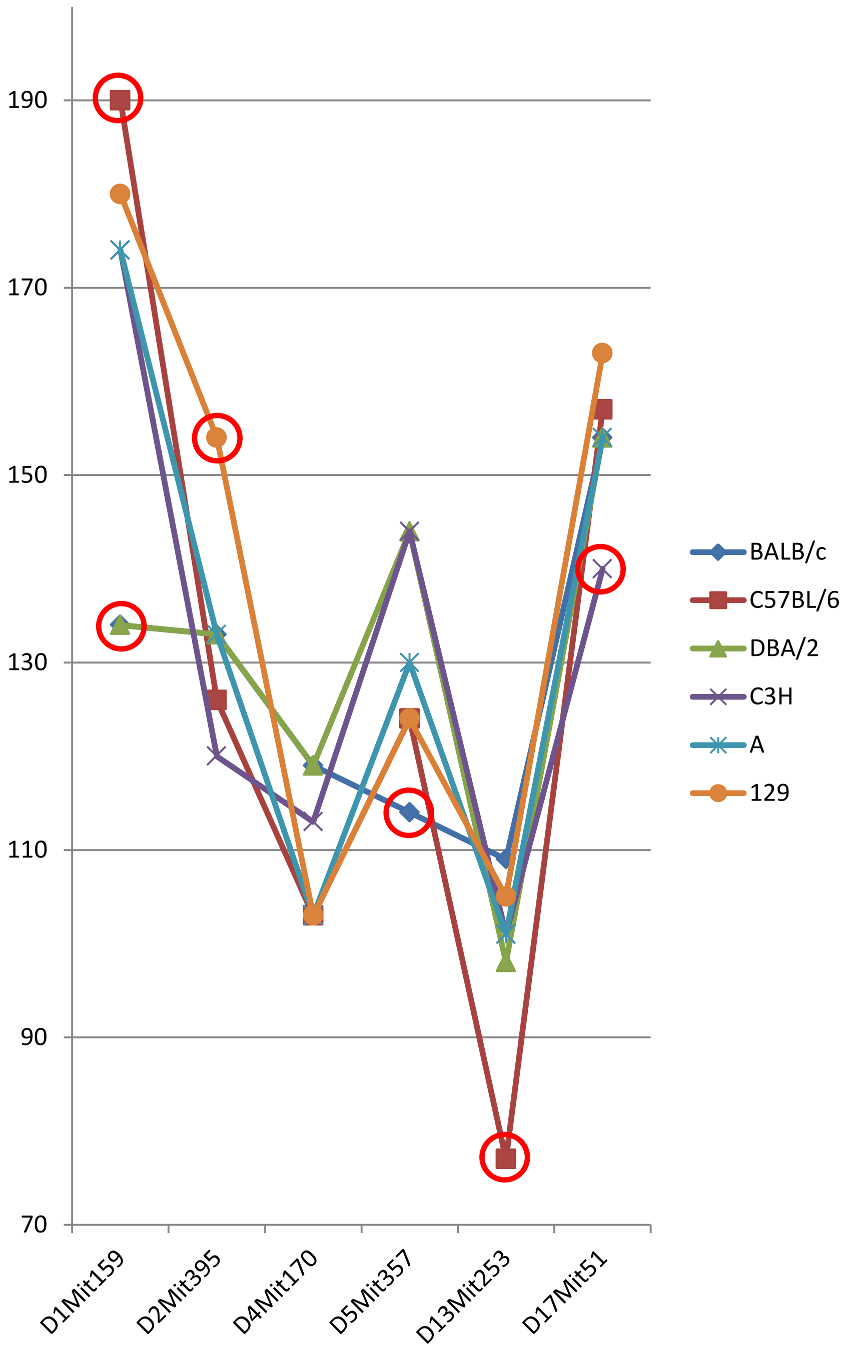

Comparison of reference SSLP profiles between 6 inbred strains. The shortest and largest sizes distinct from other strains can be markers to identify the strain, which are marked by circles.
